# Dye synthesis in the Pechmann reaction: catalytic behaviour of samarium oxide nanoparticles studied using single molecule fluorescence microscopy†
†Electronic supplementary information (ESI) available: Detailed image analysis protocol; absorbance spectra of Sm_2_O_3_NP in DMSO and in EtOH; SEM image and DLS data for supernatant containing leached particles; emission spectrum of coumarin 153 product; background scattering intensity–time trajectory; intensity–time trajectories for control TIRFM experiments; transmission image of TIRFM field of view and intensity–time trajectory corresponding to a TIRFM image sequence recorded in the presence of polydisperse Sm_2_O_3_NP; TIRFM image sequences. See DOI: 10.1039/c5sc03214h


**DOI:** 10.1039/c5sc03214h

**Published:** 2015-11-09

**Authors:** Gregory K. Hodgson, Stefania Impellizzeri, Juan C. Scaiano

**Affiliations:** a Department of Chemistry , Centre for Catalysis Research and Innovation , University of Ottawa , 10-Marie-Curie , Ottawa , Ontario K1N 6N5 , Canada . Email: Scaiano@photo.chem.uottawa.ca

## Abstract

Photochemically prepared samarium oxide nanoparticles (Sm_2_O_3_NP) efficiently catalyze the formation of coumarin 153 *via* the Pechmann trans-esterification and condensation process. The formation of the fluorescent coumarin allowed the catalytic process to be monitored in real time at the single molecule level using Total Internal Reflection Fluorescence Microscopy (TIRFM). Benchtop experiments conducted in parallel demonstrated that the observed catalysis occurred in solution rather than by pure heterogeneous catalysis and is due to a mobile population of small Sm_2_O_3_NP released from a polydisperse original sample containing larger particles. TIRFM provided unique insights by demonstrating that catalysis by these smaller colloidal particles is in fact a surface process, while the larger particles are merely suppliers of the small catalytic nanostructures. We refer to this behaviour as a semi-heterogeneous catalytic system. This work showcases the opportunity that single molecule fluorescence techniques can offer in terms of understanding and ultimately improving benchtop and scaled-up synthesis. This specific example highlights the general applicability of this approach to the study of widely-utilized chemical reactions and lays the groundwork for researchers to adopt similar strategies in other systems.

## Abstract 2

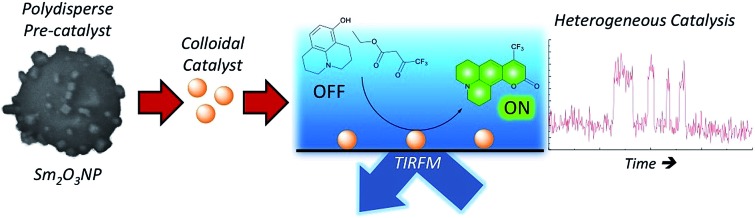
Single molecule fluorescence microscopy has shown that samarium oxide nanoparticles efficiently catalyze the formation of coumarin 153 *via* a semi-heterogeneous catalytic process.

## A. Introduction

Owing to unique physicochemical properties not observed at the macroscale, nanoparticles (NP) have found applications ranging from pharmaceuticals to optoelectronics to alternative energy sources.^1^–^4^ Catalysis often plays an integral role in these applications and evaluating the properties and the performance of a new catalytic material is essential; in particular, the true nature of the catalytic process requires attention to establish whether the process is purely homogeneous or purely heterogeneous, or both.^5^,^6^,^8^–^10^ In addition to performing heterogeneous catalysis, nanomaterials may act as precursors to homogeneous catalysis by leaching metal ions which then form catalytically active organometallic complexes in solution.^3^,^5^,^6^,^8^,^11^ Nanomaterials may also exhibit ‘semi’-heterogeneous behaviour, where catalysis occurs in solution but as a consequence of the release of mobile colloidal NP acting as heterogeneous catalysts (this work), or by *in situ* generation of small clusters that are the truly catalytically active species. The reverse scenario, semi-homogeneous catalysis, is also a possibility; organometallic complexes intended for homogeneous catalysis have the potential to precipitate catalytically active NP in solution.^5^,^6^,^9^,^10^ Mechanistically different, an example has recently been reported in which catalytically active copper clusters are generated *in situ*, by the sequential reduction of copper salts to copper NP to clusters.^12^ Separation and proper disposal of homogeneous catalysts can be a costly and time-consuming process, making pure heterogeneous catalysis attractive from both an industrial and an environmental perspective. Indeed, while many colloidal metal nanostructures and NP immobilized onto active or inactive matrices possess high degrees of catalytic activity and sometimes reusability, catalysts that operate on a purely heterogeneous basis present valuable advantages, such as: easier extraction of the product; minimal product contamination; facile catalyst recovery and in many cases reusability.^8^,^13^ Reliably identifying the mode of catalysis is therefore a priority during the development of new nanostructured catalytic materials. Unfortunately, current methods of distinguishing between homogeneous and heterogeneous catalysis leave room for improvement. For example, control experiments can be conducted using supernatant obtained by washing the catalyst with solvent.^11^ Although such tests may be effective on a per-case basis, they do not provide any additional insight regarding the underlying catalytic mechanism. A bottom-up approach to catalyst design may benefit from a more generally applicable means of not only detecting the mode of catalysis, but understanding the behaviour of new catalytic materials at the single molecule level.

Powerful imaging techniques traditionally employed in the biological sector are becoming increasingly available to chemists,^2^,^3^,^5^,^6^,^14^–^20^ providing continuity between the bench scale and single molecule level. Such techniques include Total Internal Reflection Fluorescence Microscopy (TIRFM), Fluorescence Correlation Spectroscopy, and Fluorescence Lifetime Imaging Microscopy. Incorporating these tools into catalysis research can shed light on the spatiotemporal heterogeneities often displayed by solid catalysts, thereby adding a new dimension to catalyst design.^2^,^3^,^17^,^19^ In addition to identifying the nature of the catalysis directly, catalysts’ dynamics at the single molecule level can provide a valuable link between their physicochemical properties, catalytic mechanisms and their applicability on an industrial scale. This strategy of correlating macroscopic catalytic performance with single molecule studies (to which we have referred to as ‘*from the mole to the molecule*’)^6^,^15^ allows for an iterative design process enabling rational design of materials with targeted reactivity and customized combinations of desirable properties. Using single molecule techniques to improve benchtop and scale-up strategies (or ‘*from the molecule to the mole*’) we foresee a future where improved synthetic procedures are the direct result of mechanistic details obtained in single molecule studies.

Milder reaction conditions and potential catalyst reusability are attractive aspects of heterogeneous or semi-heterogeneous catalysis. Nanocatalysts can offer alternatives to the harsh experimental conditions often encountered in traditional homogeneous catalysis.^9^,^13^ Coumarin derivatives are routinely synthesized *via* the Pechmann trans-esterification and condensation reaction. The experimental protocol generally requires elevated temperatures and stoichiometric quantities of strongly acidic homogeneous catalysts.^21^–^23^ We recently reported on the photochemical synthesis and characterization of novel samarium oxide nanoparticles (Sm_2_O_3_NP) possessing Brønsted acidity.^24^ We show here that Sm_2_O_3_NP can efficiently catalyze the formation of coumarin 153 *via* the Pechmann reaction and focus on the use of TIRFM to establish the mode of catalysis at work.

## B. Experimental

### Methods

Except where otherwise noted, all reagents and solvents were obtained from either Sigma Aldrich or Fisher Scientific. Sm_2_O_3_NP were photochemically prepared and characterized according to reported protocols.^24^ The Pechmann trans-esterification and condensation reaction was carried out using a 1 : 2 molar ratio of 8-hydroxyjulolidine (**1**) to ethyl 4,4,4-trifluoroacetoacetate (**2**). This amounted to 0.211 mmol of the latter and 0.106 mmol of limiting reagent. These reagents were added to a 10 mL round bottom flask along with a magnetic stir bar, 1.5 mL of 99% EtOH and an appropriate mass of Sm_2_O_3_NP. Reagents **1** and **2** as well as the coumarin 153 product (**3**) used for control experiments were obtained from Sigma-Aldrich and used without further purification. The flask was fitted with a condenser to prevent solvent evaporation. The reaction proceeded for 24 h at 65 °C (or room temperature, for certain control reactions) while stirring at approximately 500 rpm. For the control reaction carried out under LED irradiation (465 nm, 130 mW, LED ENGIN), the experiment was performed in a 3 mL quartz cuvette at room temperature while stirring. The LED is equipped with a SynJet ZFlow 65 cooler (Nuventix) coupled to a heat sink (24W PAR20, Nuventix) to prevent heating the solution during the reaction. In some cases, the results of which are summarized in Fig. 2, 3 mg of Sm_2_O_3_NP were first added to 1.5 mL 99% EtOH in a 10 mL flask equipped with a condenser and were stirred at 65 °C for 24 h. The resulting solution was centrifuged for 30 min at 3000 rpm (Drucker Co., Horizon model). The orange supernatant was extracted and used to perform the Pechmann reaction by adding 1 eq. of **1** and 2 eq. of **2** according to the protocol described above. In all cases, isolated yields of **3** were obtained by preparative TLC (SiO_2_, CH_2_Cl_2_ : EtOAc 9 : 1 v/v). The purified product was thoroughly washed with EtOAc and the solvent was distilled under reduced pressure (Buchi Rotovapor model R-200). After the product mass was recorded, the formation of **3** was confirmed by ^1^H NMR (Bruker AVANCE 300, CDCl_3_) and high-resolution EI mass spectrometry (HRes, Concept S1, magnetic sector mass spectrometer). Mass spectra were acquired at the John L. Holmes mass spectrometry facility at the University of Ottawa. Absorbance spectra were obtained using a Cary-50 UV-visible spectrophotometer. Dynamic Light Scattering (DLS) measurements were performed on Sm_2_O_3_NP dissolved in DMSO (2 mg mL^–1^) using a Zetasizer Nano-ZS (Malvern Instruments, 633 nm laser) at 20 °C. SEM was conducted using a JEOL JSM-7500F field emission scanning electron microscope where a drop of Sm_2_O_3_NP suspended in CH_3_CN was placed onto a carbon film coated Cu mesh grid (electron microscopy sciences model CF-400-Cu) and evaporated under ambient conditions.

### Single molecule fluorescence microscopy

TIRFM was performed with an Olympus FV1000 TIRF microscope (Olympus, Japan). Light from a CW 488 nm Ar laser was collimated and focused through a fiber-optic coupling unit before passing through a beam splitter cube (500dcxr, Chroma) and a 482/18 nm band pass filter (Semrock) which reflected the appropriate excitation light into an oil-immersion Total Internal Reflection (TIR) objective (×100, N.A. 1.45, Olympus, PLAPO). Fluorescence emission passed back through the TIR objective, was filtered by a 525/45 nm band pass filter (Semrock) and was then focused into an EM-CCD (Rolera EM-C^2^, Q-Capture, Surrey, Canada). Single molecule TIRFM experiments were conducted by flowing a 1 : 2 equimolar solution of 1 nM **1** and **2** at 1 mL h^–1^ through a flow cell reactor (Chamlide model CF-S25-B) placed over a clean, round glass coverslip spin-coated with 50 μL of 0.05 mg mL^–1^ Sm_2_O_3_NP. The apparatus was positioned atop the objective of the TIRFM system and the sample was irradiated at *λ*_Ex_ 488 nm. Product emission was recorded by the EM-CCD at 10 frames per s. Each frame consisted of a 501 × 502 pixel (px), 80 × 80 μm image with a pixel size of 159 nm. The nature of the reaction under investigation (*i.e.* non-emissive reagents forming an emissive product) enabled selective excitation of a small number of product molecules, the formation of which could be visualized as 100 s, 1000 frame image sequences consisting of bright bursting events on a dark background (ESI Video 1†).

### Image analysis protocol

Analysis of TIRFM image sequences was carried out using a combination of ImageJ (NIH), MATLAB (MathWorks) and OriginLab software. In brief, 3 × 3 px regions of interest (ROIs) were selected based on the automated identification of stochastic emission (bursting) representing the formation of coumarin 153 product molecules. After background subtraction was performed with ImageJ (rolling ball algorithm, 10 px radius), bursting events were examined graphically. This was done by first using ImageJ to measure the mean fluorescence intensity inside each ROI for every frame in a 100 s image sequence. These data were tabulated and imported into OriginLab, where frame numbers were converted to units of time. Mean intensity was then plotted as a function of time to generate a unique intensity–time trajectory for every ROI (*e.g.*Fig. 4). Three-dimensional surface projections were constructed in ImageJ, by measuring the mean intensity of every pixel in an 80 × 80 μm focal area (the complete TIRFM field of view) for every frame in a 100 s image sequence comprised of 1000 frames. The accumulated fluorescence intensity at each pixel location was then plotted as a 3D surface projection.

## C. Results and discussion

### Bench scale evaluation of Sm_2_O_3_NP as a Brønsted acid catalyst

**Scheme 1:**
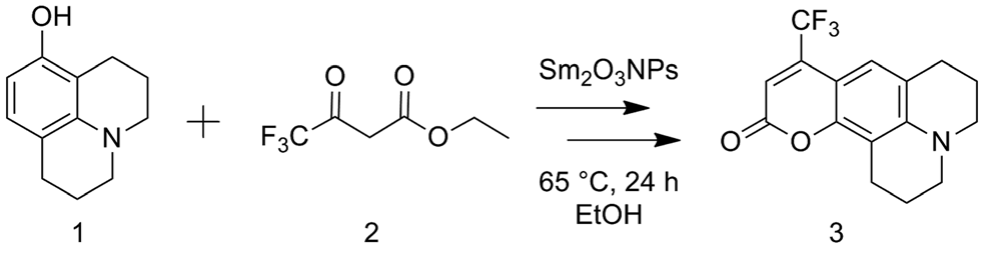
Overall reaction for the preparation of coumarin 153 *via* the Sm_2_O_3_NP-catalyzed Pechmann trans-esterification and condensation process.

The potential to utilize the Brønsted acidity of photochemically prepared Sm_2_O_3_NP for catalysis was evaluated using the reaction between 8-hydroxyjulolidine (**1**) and ethyl 4,4,4-trifluoroacetoacetate (**2**) to form coumarin 153 (**3**) as a model system (Scheme 1). The Pechmann trans-esterification and condensation reaction is a multi-step process in which a strong acid plays multiple (proton-transfer) roles.^22^ Performing the reaction for 24 h at 65 °C under air produced **3** in high yields (Table 1). The product was purified by preparative TLC and identified by ^1^H NMR and EI-MS.

**Table 1:** Results of Sm_2_O_3_NP-catalyzed formation of coumarin 153 and relevant control reactions

Catalyst	Amount of catalyst	Time (h)	Temperature (°C)	Yield of **3**
Sm_2_O_3_NP^*a*^	3 mg	24	65	93%
No catalyst	—	24	65	15%
4-HEBA	8.3 mg	68	65	34%
Sm_2_O_3_NP supernatant	1.5 mL	24	65	100%

^*a*^Reaction performed in the dark.

The yield of **3** increased from 15% in the absence of a catalyst, to 93% using a catalytic mass of Sm_2_O_3_NP (8.6 × 10^–3^ eq.). This dramatic increase in efficiency demonstrates that Sm_2_O_3_NP catalyze the reaction. Since the NP are stabilized by 4-hydroxyethoxybenzoic acid (4-HEBA), it was necessary to establish the possible catalytic role of the stabilizer in the formation of coumarin 153. As shown in Table 1, a large excess of 4-HEBA in the absence of Sm_2_O_3_NP resulted in a significantly reduced yield, even after 68 h. An isolated yield of 93% encouraged further investigation of Sm_2_O_3_NP as a heterogeneous alternative to commonly-used homogeneous strong acid catalysts.^21^–^23^ However, it became apparent that the mode of catalysis was not purely heterogeneous, as evidenced by benchtop experiments designed to probe the nature of the catalytically active species.

**Fig. 1:**
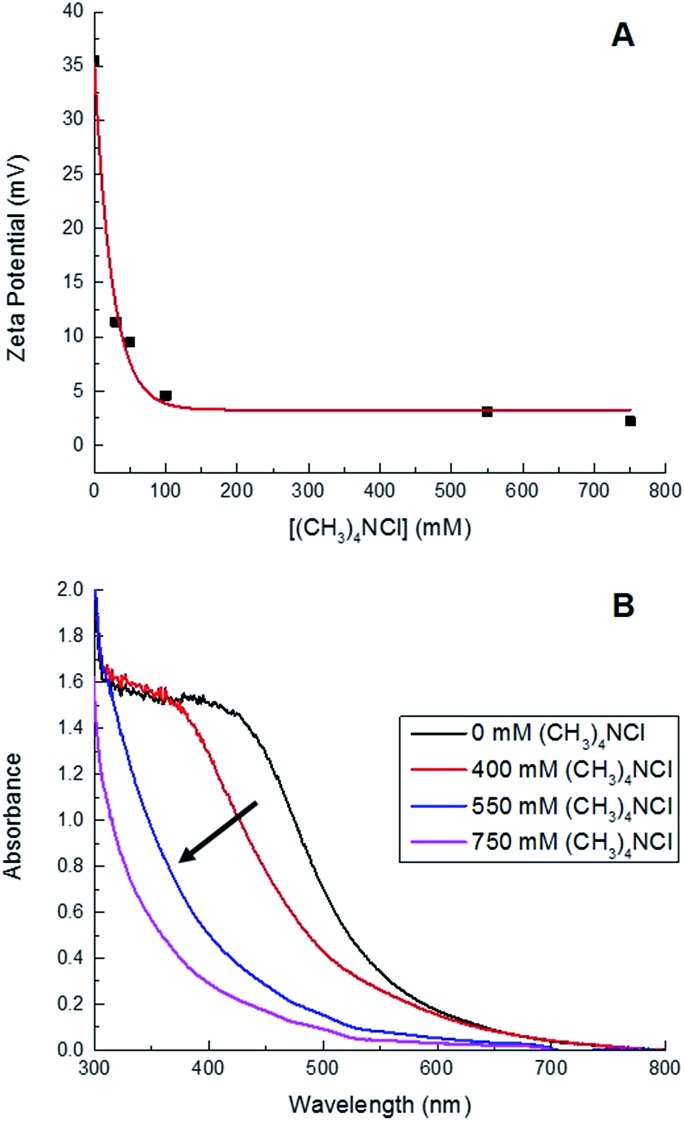
Decreasing zeta potential (A) and absorbance (B) of a solution of ≈0.2 mg Sm_2_O_3_NP dissolved in 1 mL 99% EtOH as a function of increasing ionic strength attained by adding various quantities of (CH_3_)_4_NCl.

First, Sm_2_O_3_NP were placed under conditions identical to those used for the Pechmann reaction (*i.e.* catalyst mass, solvent volume, temperature and duration) but in the absence of **1** and **2**. Centrifuging the resulting mixture at 3000 rpm produced a Sm_2_O_3_NP pellet and an orange supernatant resembling the colour of Sm_2_O_3_NP dissolved in DMSO. The absorbance spectra of these two orange solutions overlapped well, and SEM imaging confirmed the suspected presence of NP in the supernatant (Fig. S1 and S2, ESI†). It has previously been reported that the solid Sm_2_O_3_NP are polydisperse, ranging in size from approximately 70–700 nm.^24^ As would be expected, those observed in the supernatant were at the low end of this range. Dynamic Light Scattering (DLS) suggested a mean hydrodynamic diameter less than 150 nm (Table S1, ESI†). Using this supernatant as both the solvent and the catalyst in the Pechmann reaction produced a quantitative yield of **3** in 24 h (Table 1). Further, it was found that just 0.2 mg of colloidal Sm_2_O_3_NP were responsible for this yield, indicating that the catalytically active species released from the polydisperse sample of Sm_2_O_3_NP constituted just 6.7% of the total mass of solid. A related example was recently reported in which copper nanoparticles release smaller particles or clusters that are ultimately responsible for the catalysis.^12^ Surprisingly, the smaller Sm_2_O_3_NP particles remained stable in EtOH even after centrifuging at 10 000 rpm for 30 min at room temperature, followed by a second centrifugation at 10 000 rpm for 30 min at 4 °C. Their high colloidal stability relative to the larger Sm_2_O_3_NP is reflected in the zeta potential of the particles remaining in solution which, at +35 mV, is significantly higher than that of polydisperse Sm_2_O_3_NP in DMSO (+23 mV).^24^ Interestingly, increasing the ionic strength of the solution provided a convenient means of catalyst separation. The zeta potential of colloidal particles is inversely proportional to ionic strength, and the zeta potential at the isoelectric point is zero.^25^ Increasing the ionic strength of the solution provides counterions that repress the electric double layer surrounding colloidal particles and leads to decreased particle–particle repulsion, thus destabilizing the system and favouring particle aggregation. Here, the addition of (CH_3_)_4_NCl resulted in decreased zeta potential and a greater propensity for both spontaneous and centrifuge-assisted NP precipitation (Fig. 1). The Sm_2_O_3_NP began to precipitate, upon centrifugation at 10 000 rpm for 15 min, when the zeta potential reached +9.5 mV (50 mM (CH_3_)_4_NCl). As shown in Fig. 1, the absorbance of the solution followed a similar trend, decreasing with zeta potential and increased ionic strength.

**Fig. 2:**
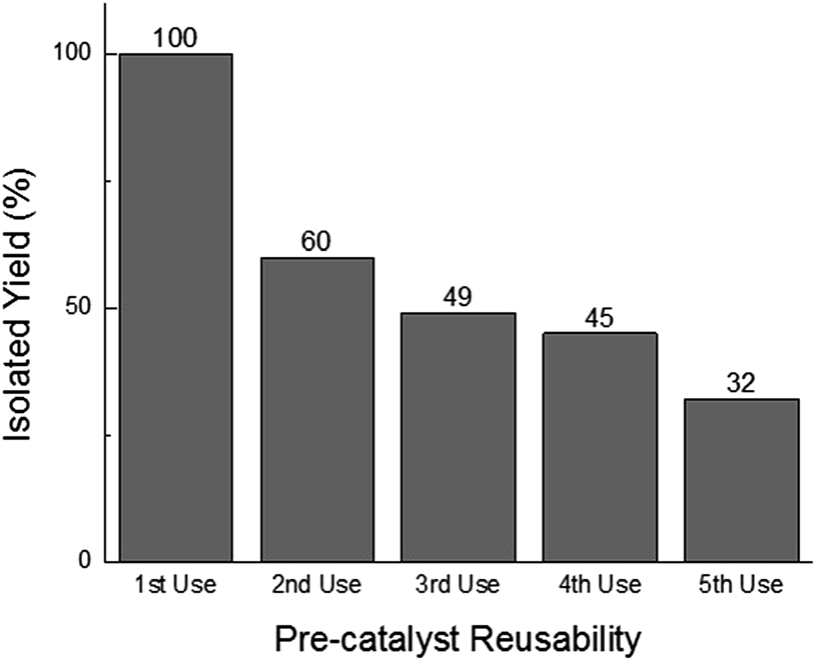
Reusability of the solid Sm_2_O_3_NP pre-catalyst. Each usage represents the isolated yield of coumarin 153 obtained by preparative TLC after performing the reaction between **1** (1 eq.) and **2** (2 eq.) at 65 °C for 24 h in the supernatant obtained by centrifuging a sample of 3 mg Sm_2_O_3_NP previously stirred for 24 h at 65 °C in 1.5 mL 99% EtOH.

**Fig. 3:**
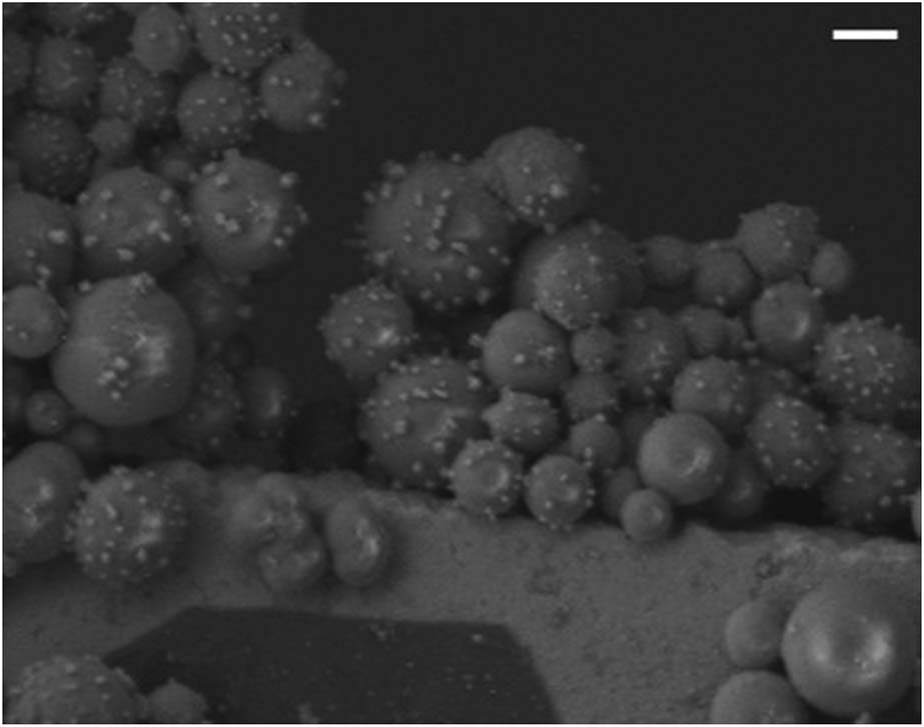
Representative SEM image demonstrating that some of the small catalytic Sm_2_O_3_NP, which become colloidal particles during the reaction, are already present in the original polydisperse pre-catalytic powder. Note that the sizes of the particles shown above are in good agreement with DLS performed upon supernatant containing catalytically active colloidal particles. Scale bar is 1 μm.

In addition to offering ease of separation, non-homogeneous catalysts can often be reused several times. In this case the solid polydisperse Sm_2_O_3_NP can function as a pre-catalyst, providing a continuous source of catalytically active colloidal Sm_2_O_3_NP over several rounds of catalysis. As shown in Fig. 2, repeatedly exposing and recovering a single 3 mg sample of the pre-catalyst provided a fresh supply of catalytically active colloidal Sm_2_O_3_NP, with the isolated yield of **3** steadily decreasing as the smaller Sm_2_O_3_NP were diminished from the polydisperse sample of particles. Electron microscopy confirmed the presence of such particles in the original polydisperse sample (Fig. 3).

Although the Sm_2_O_3_NP represent a separable, reusable catalyst for the formation of **3**, the question of the exact mode of catalysis remained unclear. Whereas leaching into solution or even proton leaching by colloidal Sm_2_O_3_NP could result in homogeneous catalysis, heterogeneous catalysis performed by separable, colloidal Sm_2_O_3_NP released from solid polydisperse Sm_2_O_3_NP would be better described as a semi-heterogeneous process. Benchtop experimentation demonstrated that a subpopulation of the polydisperse Sm_2_O_3_NP do form a stable colloidal suspension during the reaction, but was unable to unequivocally establish whether or not the catalysis occurs on the surfaces of the smaller, mobile Sm_2_O_3_NP. We therefore turned to single molecule fluorescence microscopy in an effort to gain a more in-depth understanding of the catalytic mechanism involved in this system.

### Investigating the catalytic behaviour at the single molecule level

In order to identify the mode of catalysis observed at the bench scale, the Sm_2_O_3_NP-catalyzed preparation of coumarin 153 was monitored at the single molecule level using TIRFM. This technique benefits from enhanced axial and lateral resolution provided by an evanescent field propagating parallel to the sample surface. The field is strongest at the interface between the glass coverslip and the reaction medium and decreases exponentially with distance. This effectively generates an excitation source in the form of a thin film allowing a small number of molecules to be selectively excited. Together with low sample concentration, this increases the signal-to-noise ratio by eliminating secondary fluorescence from molecules situated outside the primary focal plane.^2^,^6^ In an appropriately designed system, TIRFM enables real-time monitoring of catalysis at the single molecule level and with single molecule sensitivity.^2^,^3^,^5^–^7^,^14^–^20^

The catalytic preparation of coumarin 153 induces fluorescence emission centred at 532 nm (Fig. S3, ESI†). Although the Pechmann reaction is most efficient at temperatures exceeding 65 °C (in EtOH), it was necessary to work within the limits of the instrumentation used for fluorescence microscopy. Nonetheless, even at room temperature, enough product formation could be observed to monitor the catalysis using TIRFM (Table S2, ESI†). Further, the number of catalytic events that can be observed in a short time is enhanced by continuously supplying a fresh source of reagents by flowing **1** and **2** atop the catalyst at a rate of 1 mL h^–1^ during TIRFM experiments. An additional control reaction performed at room temperature under irradiation with a 130 mW 465 nm LED provided assurance the catalysis is not affected by the cw laser irradiation (488 nm, 5 mW) used to excite **3** for TIRFM (Table S2, ESI†).

Product formation was visualized in the form of 100 s image sequences each showing stochastic fluorescence bursting on a dark background (ESI Video 1†). Identifying the locations of bursting events and subsequently plotting fluorescence intensity as a function of time can allow for accurate discrimination between heterogeneous and homogeneous catalysis.

**Fig. 4:**
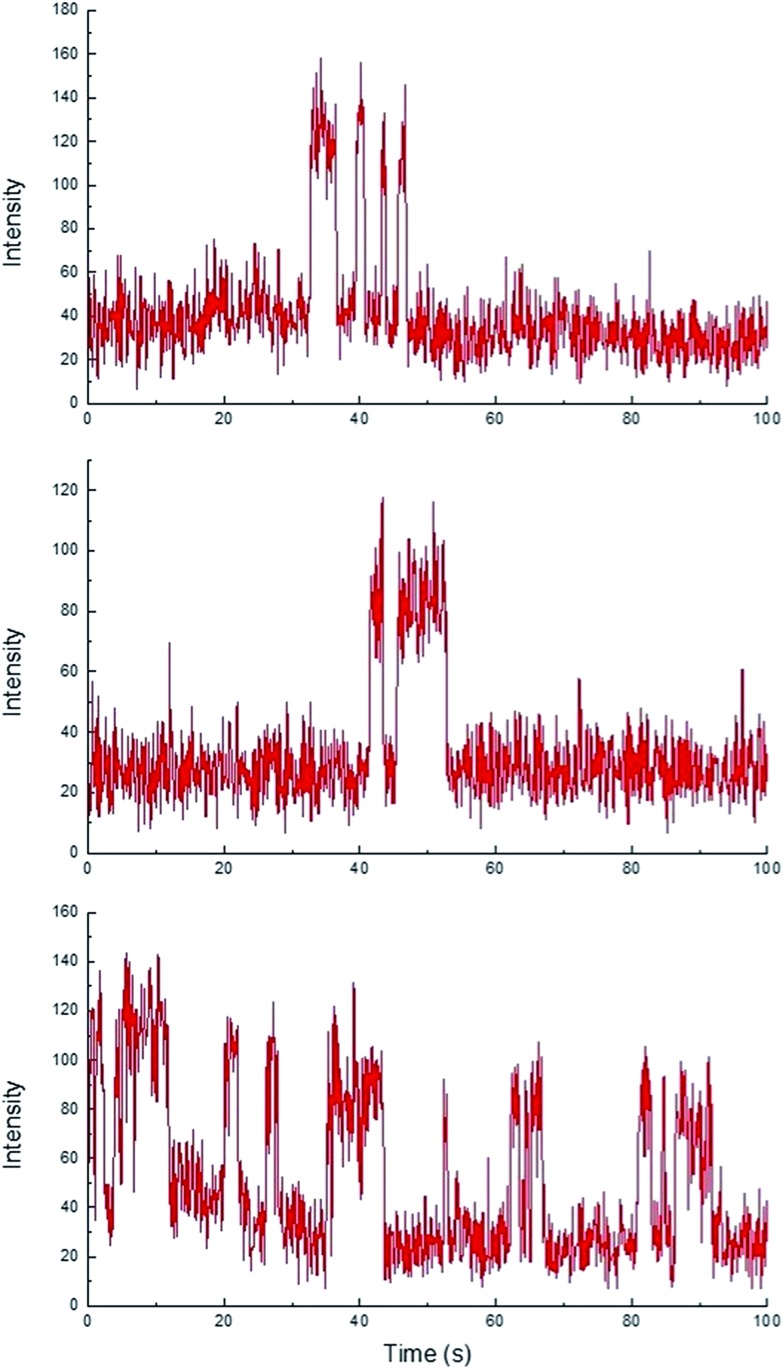
Representative intensity–time trajectories showing the intensity profile and duration of repetitive fluorescence bursts occurring at three different 3 × 3 px ROIs over 100 s, 1000 frame TIRFM image sequences obtained at room temperature. Note that the individual bursting events have roughly the same intensity, each representing emission from a single molecule.

While homogeneous or non-catalytic product formation would lead to randomly distributed single bursting events as molecules of **3** form in solution and randomly diffuse in and out of the focal plane, heterogeneous catalysis is characterized by repetitive bursting in discrete locations, as a result of molecules of **3** repeatedly being formed on the surfaces of static catalytic particles before diffusing out of the field of view.^6^,^7^ As shown in Fig. 4, repetitive bursting was indeed observed when supernatant containing only the small, catalytically active Sm_2_O_3_NP was spin-coated onto a microscope coverslip and the reaction monitored using TIRFM. These bursting events are clearly distinguishable from background scattering when compared to intensity–time trajectories extracted from TIRFM image sequences recorded while flowing only solvent atop the catalyst (Fig. S3, ESI†). Moreover, no such repetitive bursting could be observed in the absence of Sm_2_O_3_NP; TIRFM image sequences recorded while flowing **1** and **2** atop a clean coverslip contained only short-lived, singular bursting events corresponding to non-catalytic product formation (Fig. S4, ESI†). It follows that the catalytic formation of **3** mediated by the small, colloidal Sm_2_O_3_NP is a surface process. This conclusion rests on the assumption that the catalytic particles contributing to the observation of repetitive bursting are essentially immobile on the time scale of a TIRFM experiment, remaining within the dimensions that define a fixed ROI (3 × 3 px here). If this were not the case, then the combined effects of low reagent concentration, low yield at room temperature and the short time scale of a TIRFM experiment would render it extremely unlikely that freely diffusing particles would independently and repeatedly arrive at the precise coordinates of a static ROI and ultimately cause multiple bursting events to be observed at that exact location. Therefore, the lateral mobility of the catalytic particles contributing to repetitive bursting does not exceed 477 nm (3 px) in this system.

**Fig. 5:**
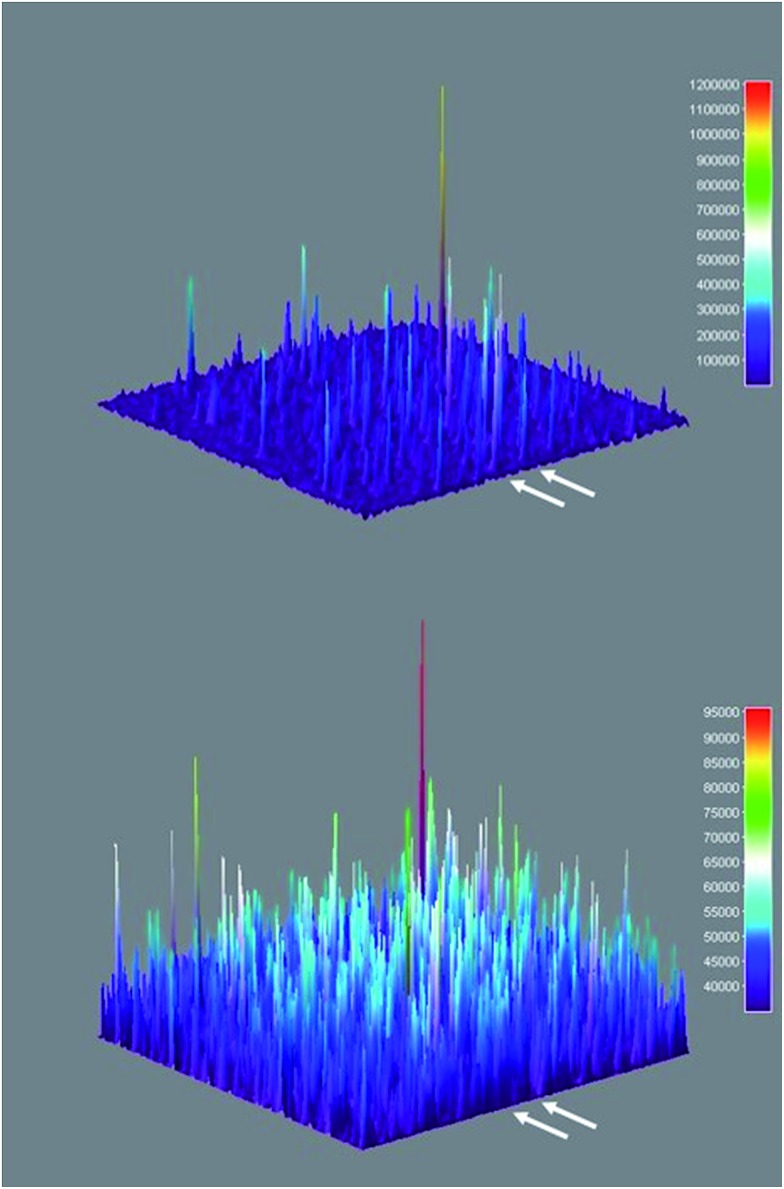
Three-dimensional surface projections showing accumulated fluorescence intensity, extracted from TIRFM image sequences recorded while flowing a 1 : 2 equimolar solution of **1** and **2** atop a microscope coverslip spin-coated with supernatant obtained after centrifuging a sample of 3 mg Sm_2_O_3_NP previously stirred for 24 h at 65 °C (upper panel) and atop a clean coverslip in the absence of Sm_2_O_3_NP (lower panel). White arrows denote positions at which 54 px (8.6 μm) wide cross-sections displayed in Fig. S5† were extracted. The full colour rendering of Fig. 5, available online, provides the clearest possible distinction between the relative intensities and distributions of the numerous peaks.

Three-dimensional surface projections of TIRFM image sequences provided efficient and immediate visual confirmation of heterogeneous catalysis by colloidal Sm_2_O_3_NP at the single molecule level, by illustrating that fluorescence intensity from **3** accumulates in specific locations (Fig. 5 upper panel). In contrast, the three-dimensional surface projection of the TIRFM experiment in which **1** and **2** were flowed atop a blank coverslip shows diffuse, lower-intensity fluorescence all over the field of view, the result of molecules of **3** forming in solution and randomly drifting in and out of the focal plane (Fig. 5 lower panel). The difference between this randomly distributed fluorescence and the buildup of fluorescence intensity in discrete locations as a result of heterogeneous catalysis on the surfaces of stationary Sm_2_O_3_NP is further exemplified in Fig. S5 of the ESI,† through a comparison of the cross-sectional views of the two plots comprising Fig. 5.

**Fig. 6:**
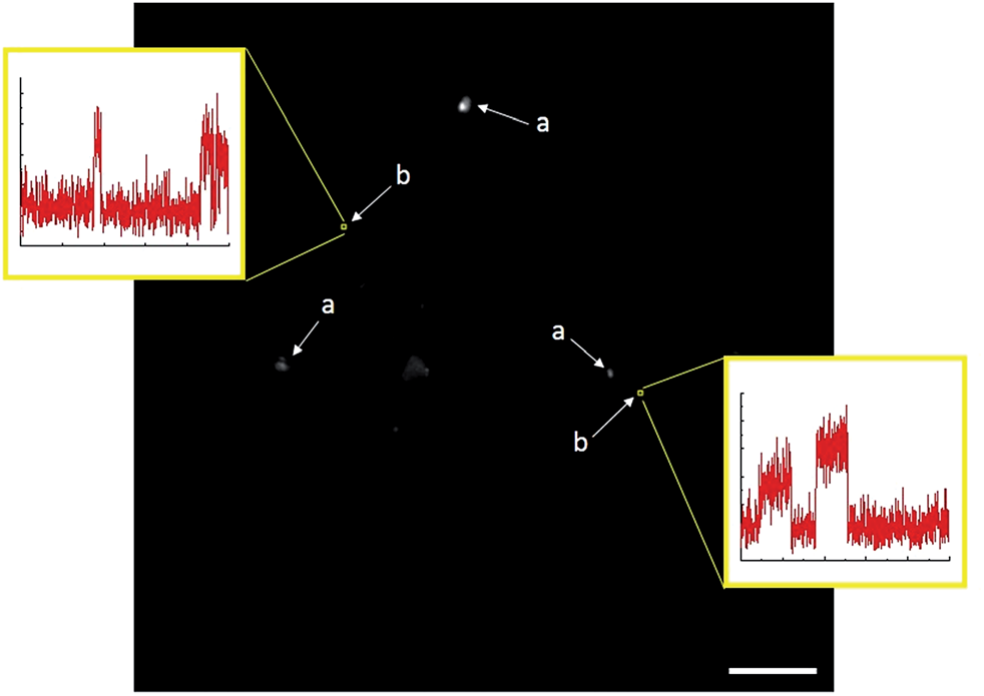
Single frame from a TIRFM image sequence recorded while flowing **1** and **2** atop a coverslip spin-coated with Sm_2_O_3_NP recovered after harvesting catalytically active colloidal Sm_2_O_3_NP four times. Large Sm_2_O_3_NP are visible due to scattering (a), and multiple bursting is only observed in 3 × 3 pixel regions where no large Sm_2_O_3_NP are located (b). Scale bar is 10 μm.

TIRFM also demonstrated that the polydisperse Sm_2_O_3_NP pre-catalyst is capable of providing a continuous supply of colloidal heterogeneous catalyst. Intensity–time trajectories corresponding to image sequences recorded while flowing **1** and **2** atop a microscope coverslip spin-coated with solid Sm_2_O_3_NP recovered after harvesting supernatant containing catalytically active colloidal particles four times, still showed repetitive bursting (Fig. 6). As shown in Fig. 6, this occurred specifically in areas where large Sm_2_O_3_NP were not located (high levels of scattering by large Sm_2_O_3_NP render them visible in TIRFM image sequences). This indicates that even after four cycles, the pre-catalyst is still able to release catalytically active colloidal particles, which is in good agreement with bench scale experiments summarized in Fig. 2.

Using TIRFM, it was also possible to establish that the small colloidal Sm_2_O_3_NP responsible for the catalysis are present in the original polydisperse sample, and are not generated *in situ via* decomposition or reduction of the larger particles (see also Fig. 3). TIRFM image sequences recorded while flowing **1** and **2** atop a coverslip spin-coated with freshly prepared pre-catalytic Sm_2_O_3_NP also contain repetitive bursting events in discrete locations where no large particles can be identified directly in the TIRFM image sequence nor in a transmission image of the same field of view (Fig. S6, ESI†). This result confirms the presence of the catalytically active species in the initial sample, which was suspected but could not be known for certain based solely upon the wide particle size distribution inherent to the original material.^24^

## D. Conclusions

Coumarin 153 can be synthesized under mild conditions with isolated yields greater than 90% using Sm_2_O_3_NP as a reusable semi-heterogeneous catalyst. The power of TIRFM and the nature of the system under investigation (*i.e.* non-emissive reagents forming an emissive product with an appropriate excitation and emission profile) made it possible to visualize the formation of a coumarin dye at the single molecule level. Importantly, single molecule fluorescence microscopy provided insight into the mode of catalysis exhibited by Sm_2_O_3_NP which was not easily gleaned from initial benchtop experimentation. We suggest that linking single molecule experiments back to catalytic performance at the bench scale can be a powerful tool in the design of novel nanostructured materials, by unmasking details that are often hidden by ensemble-averaged measurements. That is, ‘*from the molecule to the mole*’ can be a powerful strategy in the optimization of new catalysts. In this case, monitoring the catalysis at the single molecule level augmented bench scale experimentation by identifying the mode of catalysis exhibited by the truly catalytically active species, providing conclusive evidence that the Sm_2_O_3_NP-catalyzed synthesis of coumarin 153 occurs through a semi-heterogeneous catalytic mechanism in which small colloidal NP released from a polydisperse sample of solid Sm_2_O_3_NP perform heterogeneous catalysis. At low ionic strength these colloidal NP are remarkably stable, but can be readily separated by simply increasing the ionic strength *via* addition of a suitable salt. Through this specific example, we have demonstrated that single molecule fluorescence techniques can be successfully brought to bear on real synthetic challenges at the interface between physical and organic chemistry. Applying this strategy to other common organic reactions will continue to provide feedback not available using traditional ensemble-averaged techniques and is capable of identifying areas with the potential for improved efficiency, especially in catalysis. A similar approach is currently being explored in the investigation of other catalytic systems.

## Supplementary Material

Supplementary information

Supplementary movie

## References

[cit1] Chen Y.-S., Kamat P. V. (2014). J. Am. Chem. Soc..

[cit2] Sambur J. B., Chen P. (2014). Annu. Rev. Phys. Chem..

[cit3] Buurmans I. L. C., Weckhuysen B. M. (2012). Nat. Chem..

[cit4] Gomes Silva C., Juárez R., Marino T., Molinari R., Garcia H. (2011). J. Am. Chem. Soc..

[cit5] Esfandiari N. M., Blum S. A. (2011). J. Am. Chem. Soc..

[cit6] Decan M. R., Impellizzeri S., Marin M. L., Scaiano J. C. (2014). Nat. Commun..

[cit7] Xu W., Kong J. S., Yeh Y.-T. E., Chen P. (2008). Nat. Mater..

[cit8] Davies I. W., Matty L., Hughes D. L., Reider P. J. (2001). J. Am. Chem. Soc..

[cit9] Atero V., Fontecave M. (2013). Chem. Soc. Rev..

[cit10] Chen L., Chen G., Leung C.-F., Yiu S.-M., Ko C.-C., Cherdo S., Ghachtouli S. E., Sircoglou M., Brisset F., Orio M., Aukauloo A., Fang M., Engelhard M. H., Zhu Z., Helm M. L., Roberts J. A. S. (2015). ACS Catal..

[cit11] McTiernan C. D., Pitre S. P., Scaiano J. C. (2014). ACS Catal..

[cit12] Oliver-Messuguer J., Liu L., García-García S., Canós-Giménez C., Domínguez I., Gavara R., Doménech-Carbó A., Concepción P., Leyva-Pérez A., Corma A. (2015). J. Am. Chem. Soc..

[cit13] Hudson R., Li C.-J., Moores A. (2012). Green Chem..

[cit14] Roeffaers M. B. J., de Cremer G., Libeert J., Ameloot R., Dedecker P., Bons A.-J., Bückins M., Martens J., Sels B. F., de Vos D. E., Hofkens J. (2009). Angew. Chem., Int. Ed..

[cit15] Carrillo A. I., Stamplecoskie K. G., Marin M. L., Scaiano J. C. (2014). Catal. Sci. Technol..

[cit16] de Cremer G., Sels B. F., de Vos D. E., Hofkens J., Roeffaers M. B. J. (2010). Chem. Soc. Rev..

[cit17] Hensle E. M., Blum S. A., Ristanović Z., Kerssens M. M., Kubarev A. V., Hendriks F. C., Dedecker P., Hofkens J., Roeffaers M. B. J., Weckhuysen B. M. (2013). J. Am. Chem. Soc..

[cit18] Impellizzeri S., Simoncelli S., Fasciani C., Marin M. L., Hallett-Tapley G. L., Hodgson G. K., Scaiano J. C., Marin M. L., Hallett-Tapley G. L., Impellizzeri S., Fasciani C., Simoncelli S., Netto-Ferreira J. C., Scaiano J. C. (2015). Catal. Sci. Technol..

[cit19] Easter Q. T., Trauschke V., Blum S. A. (2015). ACS Catal..

[cit20] Janssen K. P. F., de Cremer G., Neely R. K., Kubarev A. V., van Loon J., Martens J. A., de Vos D. E., Roeffaers M. B. J., Hofkens J. (2014). Chem. Soc. Rev..

[cit21] Selvakumar S., Chidambaram M., Singh A. P. (2007). Catal. Commun..

[cit22] Daru J., Stirling A. (2011). J. Org. Chem..

[cit23] Gunnewegh E. A., Hoefnagel A. J., van Bekkum H., Dong F., Jian C., Kai G., Qunrong S., Zuliang L., Upadhyay K. K., Mishra R., Kumar A. (1995). J. Mol. Catal. A: Chem..

[cit24] Hodgson G. K., Impellizzeri S., Hallett-Tapley G. L., Scaiano J. C. (2015). RSC Adv..

[cit25] Attard P., Carlson J. J., Kawatra S. K., Greenwood R. (2001). Curr. Opin. Colloid Interface Sci..

