# Supplementary material for: Dye synthesis in the Pechmann reaction: catalytic behaviour of samarium oxide nanoparticles studied using single molecule fluorescence microscopy
Source: Chem Sci. 2015 Nov 9;7(2):1314–21. doi: 10.1039/c5sc03214h (PMC5975723; doi:10.1039/c5sc03214h)
Supplement: Supplementary file 1 [file SC-007-C5SC03214H-s001.pdf]

Electronic Supplementary Material (ESI) for Chemical Science.
This journal is © The Royal Society of Chemistry 2015

Supporting Information for:

Dye Synthesis in the Pechmann Reaction: Catalytic Behaviour of Samarium Oxide Nanoparticles Using Single Molecule Fluorescence Microscopy

Gregory K. Hodgson, Stefania Impellizzeri and Juan C. Scaiano*

Department of Chemistry, Centre for Catalysis Research and Innovation, University of Ottawa, 10 Marie Curie, Ottawa, Ontario K1N 6N5, Canada

Detailed image analysis protocol for the identification of ROIs…………….…………..S2
Absorbance spectra……………………………………………………………………S5
SEM of supernatant containing Sm_2O_3NP ….…………………………………...…...S6
DLS data for supernatant containing Sm_2O_3NP……………………………….....….S7
Emission spectrum of coumarin 153……………………………………………………S8
Pechmann control reactions performed at room temperature……………………………S9
Intensity vs. time trajectory for background scattering by Sm_2O_3NP……………..…...S10
Intensity vs. time trajectories recorded in the absence of Sm_2O_3NP…………………...S11
Cross-sections of 3D surface projections………………………………………………S12
Single frame of image sequence recorded in presence of polydisperse Sm_2O_3NP…….S13
Transmission image of TIRFM field of view in presence of polydisperse Sm_2O_3NP…S13
Intensity vs. time trajectory recorded in presence of polydisperse Sm_2O_3NP…………S13
References………………………………………………………………………...…..S14

Detailed image analysis protocol for the identification of ROIs

An in-house written MATLAB script "Spectacle.m" was used to identify ROIs potentially representing real bursting events. Spectacle.m was built around a freely available MATLAB sub-routine "Localize.m" which allows one to utilize the function "LocalizerMatlab.mex64", originally written and made available by Peter Dedecker from the University of Luven (Belgium). This function was designed for the precise localization of single fluorescence emitters in super-resolution microscopy image analysis. This can be done using MATLAB or through the IgorPro graphical user interface. The Localizer function has been thoroughly tested against and has performed at least as well as similar super-resolution imaging software such as QuickPALM and RapidSTORM.[1] The advantage of Localizer is that it can be incorporated into larger MATLAB scripts such as Spectacle.m, where it can be tailored to meet the needs of other applications such as TIRFM that also require efficient and unbiased automated localization of fluorescence emitters.

Specifically, Localizer analyzes an image, or series of frames of an image stack, by using one of six localization algorithms (2DGauss, 2DGaussFixedWidth, Ellipsoidal2DGauss, IterativeMulitplication, Centroid, MLEwG) in combination with a segmentation algorithm, the Generalized Likelihood Ratio Test (GLRT), to identify the locations of possible fluorescence emission. The GLRT includes a Probability of False Alarm (PFA) parameter that was left at its default value of 25%. Similar to other super-resolution software, Localizer uses these algorithms to approximate the spatial distribution of the fluorescence emission detected from individual molecules, known as the Point Spread Function (PSF), by fitting it to a mathematical function. For our

purposes the two-dimensional Gaussian function was selected because it provides the most detailed data and highest level of accuracy, despite requiring the longest computation time. The localization algorithm requires an initial estimation of the standard deviation of the PSF. This parameter was set to 1.5 px based on an estimation made using the ImageJ 3D super-resolution plugin. In addition, this value constitutes a logical maximum standard of deviation since all ROIs consisted of 3×3 pixel areas. Localizer finds the centre of the Gaussian distribution and identifies it as the location of the signal. The portion of the output of the Localizer function utilized by Spectacle.m is a tabulated list of (x,y) coordinates in px units, corresponding to precise emitter locations. For image sequences Localizer analyzes image data on a frame by frame basis. The purpose of Spectacle.m, in addition to selecting only the (x,y) coordinates of emitters from the data output by Localizer, is primarily to remove artifacts by filtering the data such that only those locations where emission is detected in at least 3 consecutive frames (i.e. 0.3 s) are retained. Since these coordinates must then be converted to 3×3 px ROIs to facilitate efficient measurement of mean intensity vs. time using ImageJ, Spectacle.m also conducts a nearest neighbor type analysis of the remaining coordinates. It examines every single remaining coordinate and checks that no other emitter coordinates fall within a ±3 px range in both the *x*- and *y*-directions. When any such scenarios occur, Spectacle.m randomly selects only one copy and discards the others. The purpose of this secondary function is to eliminate the possibility of overlapping ROIs once the data is imported into ImageJ and converted to 3×3 px ROIs, avoiding the painstaking process of manually cross-checking the coordinates of every single ROI to ensure that overlapping ROIs are not mistakenly counted multiple times. Finally, Spectacle.m returns a list of the

remaining (x,y) coordinates which are then imported into the ImageJ ROI Manager as 3×3 px ROIs with the aid of a simple ImageJ macro written using the ImageJ macro scripting language.

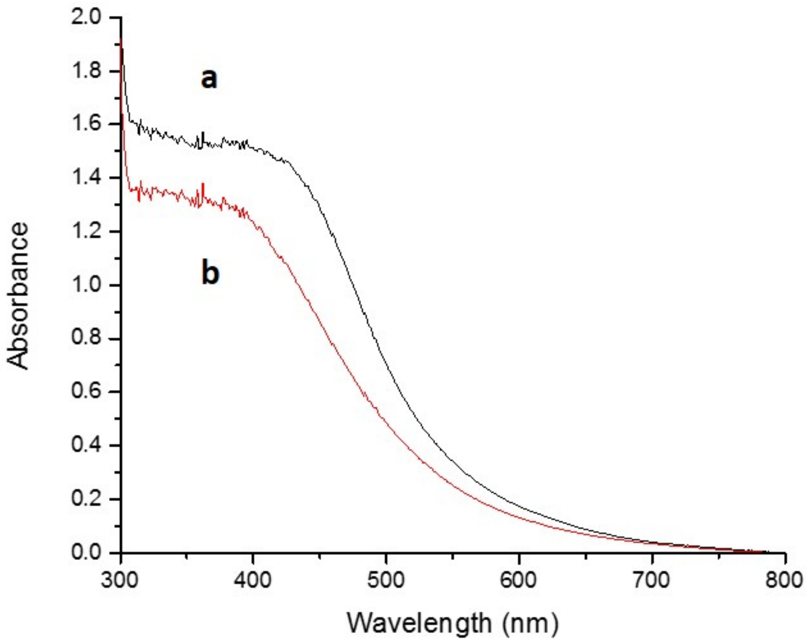

Figure S1 Absorbance spectra of: the supernatent obtained after centrifuging a sample of 3 mg Sm_2O_3NP previously stirred for 24 h at 65°C (**a**); Sm_2O_3NP dissolved in DMSO (**b**).

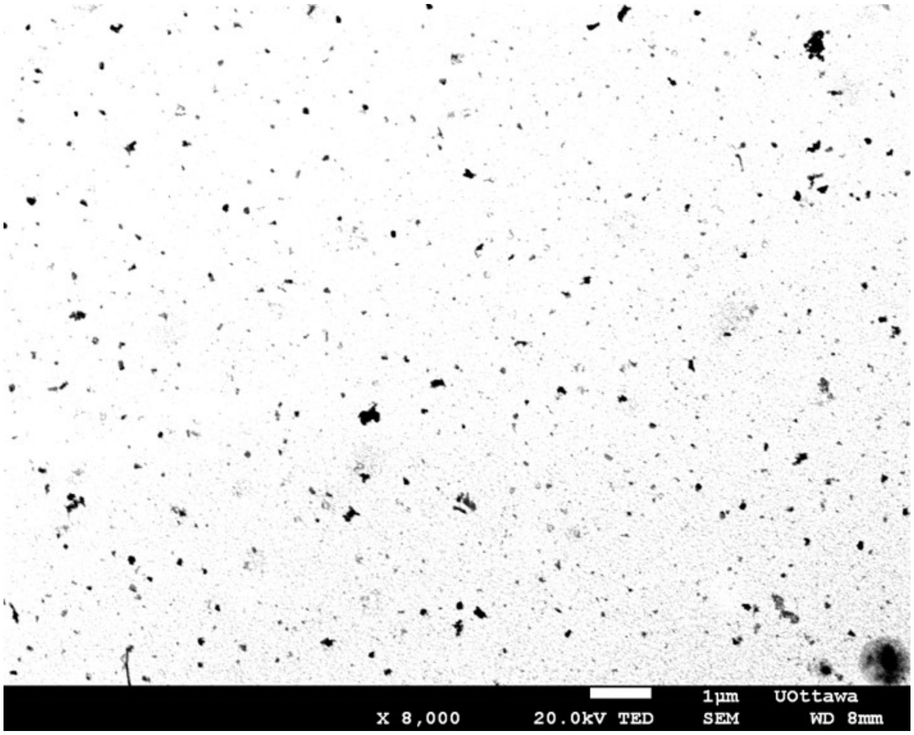

Figure S2 SEM image of the orange supernatent obtained after centrifuging a sample of 3 mg Sm_2O_3NP previously stirred for 24 h at 65°C.

Table S1 DLS data pertaining to Sm_2O_3NP present in the supernatent after centrifuging a sample of Sm_2O_3NP previously stirred in EtOH for 24 h at 65°C. All measurements were acquired at 25°C.

Measurement	Hydrodynamic Diameter Z-Average Method (nm)	Hydrodynamic Diameter Number Mean Method (nm)
1	144.0	96.28
2	143.6	81.03
3	144.2	101
4	144.6	90.23
5	142.2	87.41
6	142.5	89.29
Mean	143.5 ± 0.96	90.9 ± 6.9

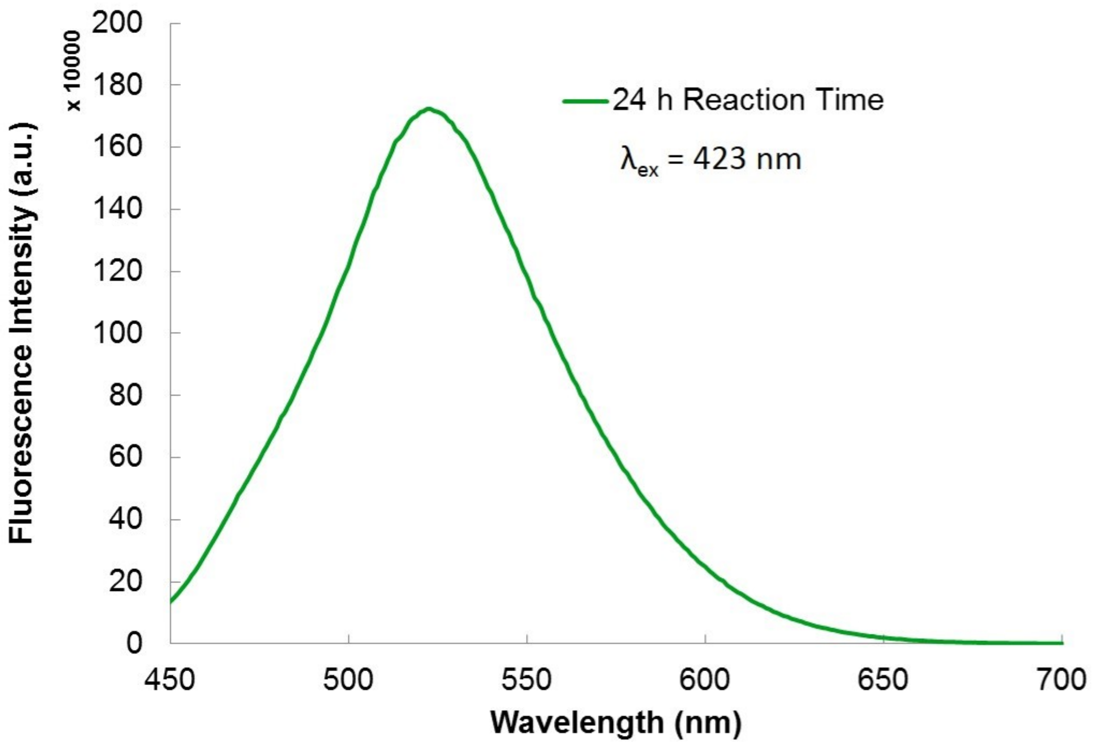

Figure S3 Fluorescence emission spectrum of coumarin 153 product obtained after 24 h reaction at 65°C in the presence of Sm_2O_3NP.

Table S2 Pechmann control reactions performed at room temperature.

Catalyst	Amount of Catalyst	Time (h)	Yield of 3
Sm_2O_3NP	3 mg	24	10%
Sm_2O_3NP[a]	3 mg	24	18%
Sm_2O_3NP supernatant	1.5 mL	24	40%
No Catalyst	-	24	5%

[a] *Reaction irradiated by a 465 nm, 130 mW blue LED*

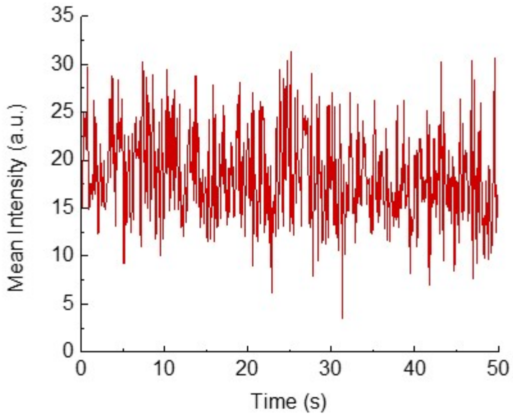

Figure S3 Representative background intensity vs. time trajectory for a 3×3 px ROI obtained from a TIRFM image sequence where solvent only was flowed over Sm_2O_3NP.

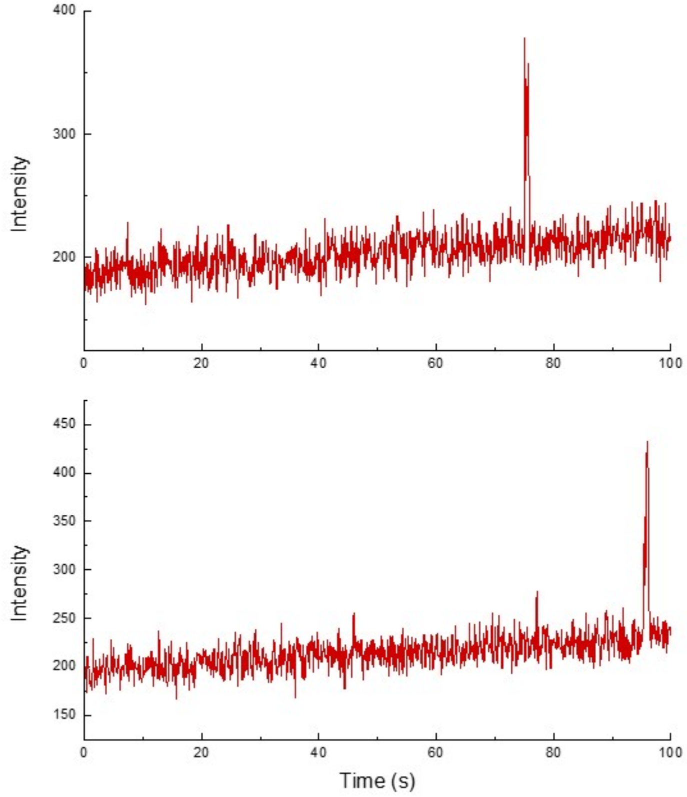

Figure S4 Representative intensity-time trajectories containing only singular bursting events, extracted from TIRFM image sequences recorded while flowing **1** and **2** in the absence of Sm_2O_3NP (i.e. atop a clean glass coverslip).

The following three-dimensional surface projection cross-sections illustrate the difference between low-intensity random (lower panel) and high intensity non-random (upper panel) distributions of accumulated fluorescence detected by recording TIRFM image sequences while flowing reagents in the absence and presence of catalytic Sm_2O_3NP, respectively. The localization of fluorescence bursting in the upper panel represents catalytic product formation on the surfaces of Sm_2O_3NP that remain essentially immobile on the time scale of a TIRFM experiment.

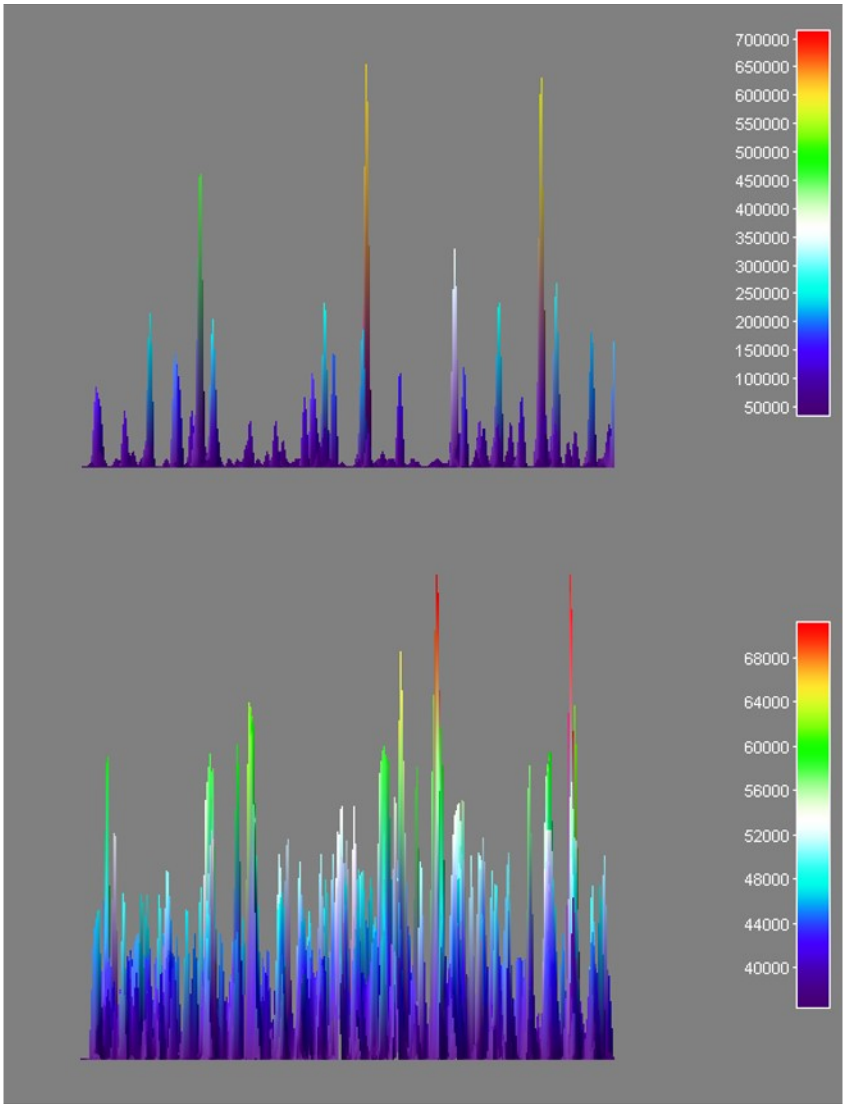

Figure S5 Side-views of 54 px, 8.6 μm wide cross-sections of the three-dimensional surface projections displayed in Figure 5 of the main text, corresponding to TIRFM experiments in which a 1:2 equimolar solution of **1** and **2** was flowed at 1 mL/h atop a microscope coverslip spin-coated with supernatant obtained after centrifuging a sample of 3 mg Sm_2O_3NP previously stirred for 24 h at 65°C (upper panel) and atop a clean coverslip in the absence of Sm_2O_3NP (lower panel). Notice the difference in the vertical scale.

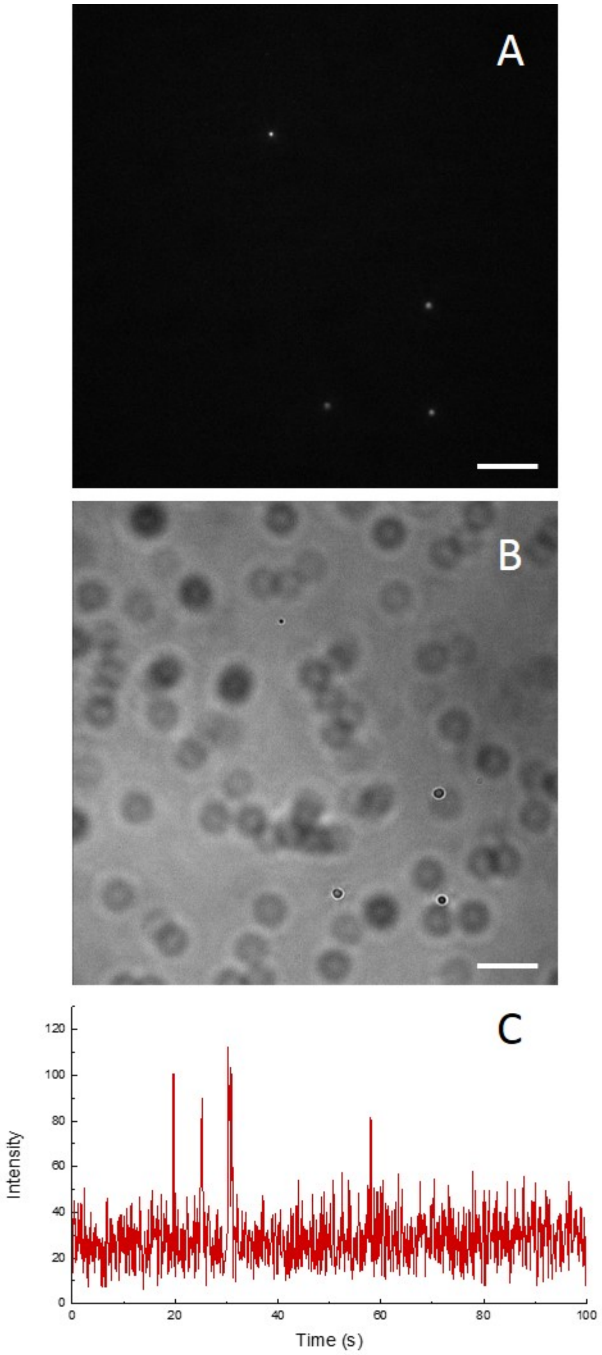

Figure S6 Single frame from a TIRFM image sequence recorded while flowing **1** and **2** atop a glass coverslip spin-coated with the original polydisperse, pre-catalytic Sm_2O_3NP (A); corresponding transmission image of the same field of view shown in A, demonstrating that the locations large Sm_2O_3NP are identifiable in TIRFM image sequences due to scattering (B); representative intensity-time trajectory extracted from a TIRFM image sequence described in A, showing repetitive fluorescence bursting in discrete locations as evidence of heterogeneous catalysis (C). Scale bars are 10 μm.

References

1 P. Dedecker, S. Duwe, R. K. Neely and J. Zhang, *J. Biomed. Opt.*, 2012, **17**, 126008.
